# The missing P waves in wide QRS tachycardia

**DOI:** 10.3389/fphys.2025.1732486

**Published:** 2026-01-23

**Authors:** Jiangying Mo, Xuan Jiang, Shuang Zhang, Xuping Li, Mingxian Chen

**Affiliations:** 1 Guilin Hospital of the Second Xiangya Hospital of Central South University, Guilin, Guangxi, China; 2 The Second Xiangya Hospital of Central South University, Changsha, Hunan, China

**Keywords:** ablation, atrioventricular nodal reentranttachycardia, conduction block, tachycardia, wide QRS waves

## Abstract

A 57-year-old male patient presented with paroxysmal palpitations for 2 years. The patient’s blood pressure was 120/76 mmHg; his heart rate was 149 beats/min. The presenting electrocardiogram showed a wide QRS complex tachycardia with an irregular rhythm. The RR intervals were irregular, and there were more complex QRS waves than P waves. The patient was first diagnosed with ventricular tachycardia. The ECG during sinus rhythm revealed a complete right bundle-branch block. The patient underwent an invasive electrophysiological study and was then diagnosed with atrioventricular nodal reentrant tachycardia (AVNRT). Following successful slow pathway radiofrequency ablation (located at the anterior part of the coronary sinus ostium), tachycardia was no longer inducible.

## Introduction

Atrioventricular nodal reentrant tachycardia (AVNRT) typically presents as a regular narrow-QRS tachycardia, but atypical conduction patterns or baseline bundle-branch block may obscure its supraventricular origin (Yamashita et al., 2024). When retrograde Wenckebach conduction occurs over the fast pathway, progressive VA prolongation and intermittent block can produce “missing” P waves and an apparent excess of ventricular complexes, giving rise to an irregular wide QRS tachycardia that closely mimics ventricular tachycardia (Hoffmann et al., 2024). This uncommon manifestation is easily misinterpreted without careful analysis of retrograde atrial activity or intracardiac intervals. We describe a patient whose irregular wide QRS tachycardia was initially suspected to be ventricular in origin but was ultimately diagnosed as typical AVNRT with retrograde Wenckebach, highlighting key diagnostic features of this rare presentation.

## Case presentation

A 57-year-old male presented to our hospital with a 2-year history of paroxysmal palpitations. He had no significant family history of cardiovascular disease or arrhythmias, no history of major medical illness, and was taking no medications. He had never undergone an electrophysiological study, catheter ablation, or any arrhythmia-related intervention. On admission, his blood pressure was 120/76 mmHg, and his heart rate was 149 beats per minute. The initial 12-lead electrocardiogram (ECG) demonstrated a wide QRS complex tachycardia with irregular rhythm, characterized by irregular RR intervals and a greater number of QRS complexes than visible P waves, leading to an initial working diagnosis of ventricular tachycardia.

Baseline laboratory tests, including complete blood count, serum electrolytes, renal and hepatic function, were all within normal limits. Transthoracic echocardiography revealed normal cardiac chamber size and preserved systolic function without regional wall motion abnormalities. A subsequent ECG obtained during sinus rhythm showed complete right bundle-branch block (RBBB).

## Interpretation and clinical course

**FIGURE 1:**
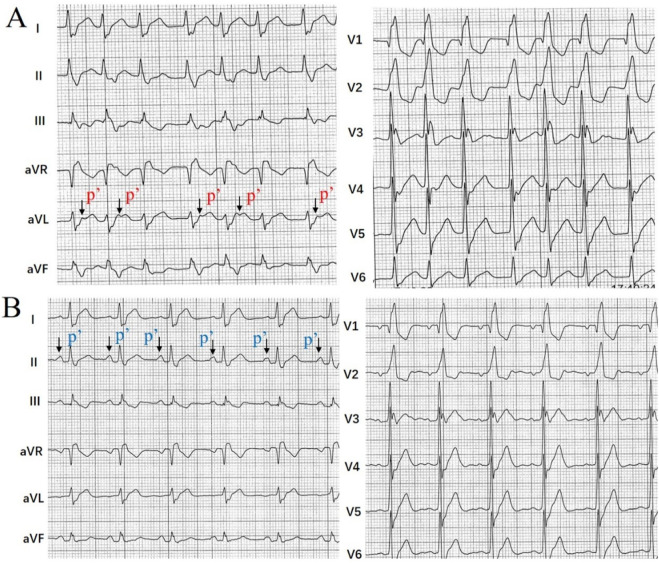
ECG presentation: **(A)** 12-lead electrocardiogram (ECG) obtained on admission. The ECG shows a tachycardia with wide QRS complexes. The RR intervals were irregular, and the retrograde P waves were gradually prolonged and appeared intermittently. There are more complex QRS waves than retrograde P waves, and the retrograde P waves were dropped on the surface ECG. The test indicated that the ventricular waves and atrial waves were dissociated. **(B)** Surface ECG during sinus rhythm.

In this patient with paroxysmal tachycardia, the tachycardia ECG showed the wide QRS complexes (RBBB) tachycardia with irregular rhythm. The RR intervals were irregular, and the retrograde P waves appeared intermittently. There are more complex QRS waves than retrograde P waves, and the retrograde P waves were dropped on the surface ECG (Figure 1A). The test indicated that the ventricular waves and atrial waves were dissociated. Based on the provided ECG (Figure 1B) showing a wide QRS complex tachycardia with an irregular rhythm, the two primary diagnostic considerations are ventricular tachycardia (VT) and atrial fibrillation (AF) with aberrancy. The wide QRS complexes and the apparent presence of more QRS waves than visible P waves (suggesting AV dissociation) support VT, while the irregularly irregular RR intervals favor AF. However, key findings argue against both: the irregularity demonstrates a patterned Wenckebach periodicity rather than chaos, and careful analysis reveals subtle retrograde P waves.

**FIGURE 2:**
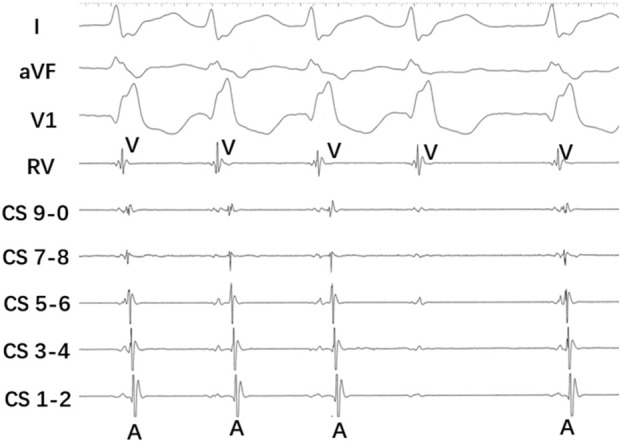
A simultaneous recording (top to bottom panel) of surface lead I, aVF, V1, and intracardiac electrograms from the coronary sinus (CS) and the right ventricular apex (RVA) was made at a paper speed of 100 mm/s. The Wenckebach phenomenon was observed. A progressive prolongation of VA conduction (increasing VA interval) with each successive beat until an A wave fails to conduct to the ventricles resulted in a dropped P wave. After the dropped beat, the cycle repeats.

On day 2, the patient was taken to the catheterization laboratory for an electrophysiological study as part of the further evaluation. The intracardiac intervals were normal during sinus rhythm. Dual AV nodal physiology, with a single jump of 66 ms in the atrial-to-His (AH) interval, was observed. The rapid, irregular A waves were not observed after tachycardia induction. During the tachycardia, progressive prolongation of the His-to-atrial (HA) interval before the blocked atrial electrogram was observed. Intermittent P waves were visible on the lead III and aVL of surface ECG during the tachycardia with retrograde block. Figure 2 shows a simultaneous recording (top to bottom panel) of surface lead I, aVF, V1, and intracardiac electrograms from the coronary sinus (CS) and the right ventricular apex (RVA) recorded at a paper speed of 100 mm/sec. The Wenckebach phenomenon was observed, characterized by a progressive prolongation of VA conduction (increasing VA interval) with each successive beat until an A wave fails to conduct to the ventricles, resulting in a dropped P wave. After the dropped beat, the cycle repeats. After the administration of intravenous isoproterenol at 1 μg/min, the VA interval during AVNRT was shortened to 50 ms.

The patient was diagnosed with typical AVNRT with retrograde conduction block of the “fast” pathway. After successful slow pathway radiofrequency ablation (located at the anterior part of the coronary sinus ostium), tachycardia was no longer inducible.

**FIGURE 3:**
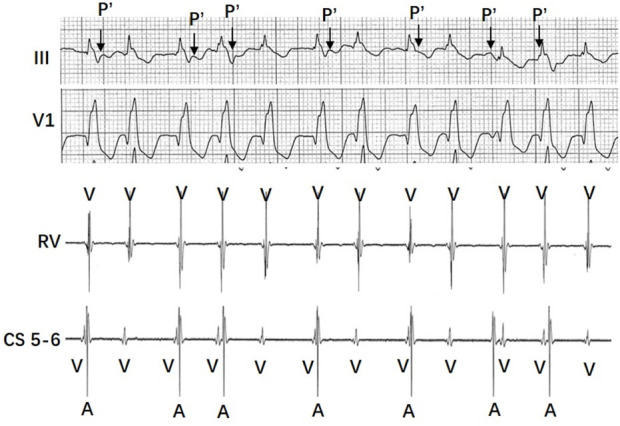
A simultaneous recording (top to bottom panel) of surface lead III, V1, and intracardiac electrograms from the right ventricular apex (RVA) and the coronary sinus (CS) was made at a paper speed of 25 mm/s. The surface ECG shows more V waves than A waves, with a rapid and widened QRS rhythm that may be mistaken for ventricular arrhythmia. However, the RR intervals are irregular. When combined with intracardiac electrograms, the findings indicate that the wide QRS tachycardia is actually AVNRT with Wenckebach (Mobitz type I) conduction over the fast pathway.

In Figure 3, the surface ECG shows more V waves than A waves, with a rapid and widened QRS rhythm that may be mistaken for ventricular arrhythmia. However, the RR intervals are irregular. When combined with intracardiac electrograms, the findings indicate that the wide QRS tachycardia is actually AVNRT with Wenckebach (Mobitz type I) conduction over the fast pathway.

**FIGURE 4:**
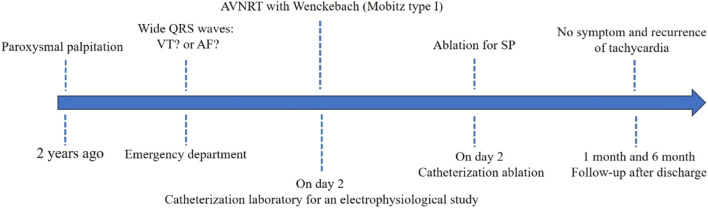
Timeline of wide QRS waves in diagnosis, management, and follow-up.

A three-dimensional anatomical map of Koch’s triangle was created using a multielectrode catheter (CARTO, Biosense Webster). At the target site, the local electrogram showed a ventricular signal with a small far-field atrial component. Radiofrequency ablation (power: 30–40 W; temperature limit: 50 °C) at this site produced an accelerated junctional rhythm. Following slow pathway modification, AVNRT was no longer inducible with programmed electrical stimulation under isoproterenol infusion. A 30-min observation period was observed prior to concluding the procedure. The patient was discharged 24 h post-procedure and remained asymptomatic with no recurrence of tachycardia at the 1- and 6-month follow-ups (timeline shown in Figure 4).

## Discussion

AVNRT is classically characterized by reentry within the dual-pathway atrioventricular (AV) nodal system, involving antegrade conduction through the slow pathway and retrograde conduction through the fast pathway (Marine, 2007). While AV block during AVNRT has been reported in 4%–10% of cases, most are antegrade block, whereas retrograde Wenckebach block is far less frequently observed (Ching Man et al., 1996; Philibert and Laurent, 2020). The mechanisms underlying such retrograde block remain a subject of electrophysiological interest and debate, particularly regarding whether the block occurs within the retrograde fast pathway, the upper common pathway, or more proximally within the atrial myocardium.

Prior reports, including the case described by Miles et al., demonstrated that increases in tachycardia cycle length or changes in autonomic tone may provoke a 2:1 or higher-degree VA block, suggesting that the retrograde fast pathway may reach its refractory limit at higher rates (Miles et al., 1994). More recent discussions, such as those presented in “Anterograde 2:1 and Retrograde 3:2 Wenckebach Block During AVNRT,” emphasized the possibility of block occurring either in the upper common pathway or within the transitional atrial tissue. These observations highlight that VA block patterns during AVNRT are not uniform and may reflect rate-dependent refractoriness in different components of the reentrant circuit (Bauernfeind et al., 1978; Okishige et al., 1992).

In our case, the hallmark finding was the presence of a retrograde Wenckebach pattern, characterized by progressive HA interval prolongation culminating in a non-conducted atrial electrogram. This pattern strongly supports a Mobitz type I block occurring within the retrograde limb of the circuit, most likely within the fast pathway or upper common pathway. The restoration of 1:1 VA conduction after isoproterenol infusion further suggests that the block was functional, driven by rate-dependent refractoriness rather than structural abnormality. Such sympathetic stimulation shortens the effective refractory period of the fast pathway, allowing retrograde conduction to resume.

Clinically, retrograde Wenckebach block during AVNRT is of particular importance because it can mimic VA dissociation, a hallmark of ventricular tachycardia (Kantharia and Mittleman, 2000). In our case, the presence of a wide QRS complex due to baseline right bundle-branch block further increased the likelihood of misdiagnosis as VT. The surface ECG showed more ventricular than atrial deflections, which could easily be interpreted as AV dissociation. Careful evaluation of intracardiac electrograms was essential to reveal the true mechanism.

Compared with previously reported cases, the present case reinforces several teaching points: (1) Retrograde Wenckebach during AVNRT is uncommon but mechanistically informative, reflecting a rate-dependent functional block within the reentrant circuit. (2) Upper common pathway physiology remains relevant in explaining variable VA conduction patterns, aligning with continuing debates highlighted in prior case reports. (3) Misinterpretation of surface ECGs is common, particularly in wide QRS tachycardia, underscoring the diagnostic value of intracardiac recordings. (4) The resolution of the block with isoproterenol demonstrates the dynamic autonomic modulation of AV nodal conduction.

Therefore, this case contributes additional clinical and mechanistic insight into the spectrum of VA conduction abnormalities during AVNRT and highlights the importance of integrating surface ECG morphology with detailed intracardiac electrophysiological evaluation.

## Take-home points

1. Misdiagnosis risk: AVNRT with retrograde conduction block can easily be misdiagnosed as ventricular tachycardia due to the presence of wide QRS complexes (RBBB) and dissociation between ventricular and atrial waves on the ECG.
2. Retrograde VA block: The case demonstrated the occurrence of a retrograde VA block during tachycardia, characterized by a gradual prolongation of the HA interval before the loss of atrial recordings, leading to intermittent P waves on the surface ECG.
3. Importance of electrophysiological study: An invasive electrophysiological study is crucial for accurately diagnosing AVNRT and distinguishing it from other arrhythmias. It also aids in identifying the dual AV nodal physiology and the nature of the retrograde conduction.

## Data Availability

The original contributions presented in the study are included in the article/supplementary material; further inquiries can be directed to the corresponding author.
