# Correction: The missing P waves in wide QRS tachycardia

**DOI:** 10.3389/fphys.2026.1800162

**Published:** 2026-02-10

**Authors:** Frontiers Production Office

**Affiliations:** Frontiers Media SA, Lausanne, Switzerland

**Keywords:** ablation, atrioventricular nodal reentranttachycardia, conduction block, tachycardia, wide QRS waves

Affiliations 1 and 2 were inverted in the wrong order and have been corrected to:

1. Guilin Hospital of the Second Xiangya Hospital of Central South University, Guilin, Guangxi, China

2. The Second Xiangya Hospital of Central South University, Changsha, Hunan, China.

The original article has been updated.

